# Disease Tolerance in ‘Anaheim’ Pepper to PepGMV-D Strain Involves Complex Interactions Between the Movement Protein Putative Promoter Region and Unknown Host Factors

**DOI:** 10.3390/v17020268

**Published:** 2025-02-15

**Authors:** Cecilia Hernández-Zepeda, Judith K. Brown

**Affiliations:** 1 Unidad de Ciencias del Agua, Centro de Investigación Científica de Yucatán, A.C., Cancún 77500, Mexico; cecilia.hernandez@cicy.mx; 2 School of Plant Sciences, The University of Arizona, Tucson, AZ 85721, USA

**Keywords:** begomovirus, host-plant resistance, post-transcriptional gene silencing, recovery phenotype, tissue tropism, transcriptional gene silencing

## Abstract

Pepper golden mosaic virus (PepGMV) is a bipartite begomovirus of pepper and tomato from North America. In ‘Anaheim’ pepper plants PepGMV-Mo strain (Mo) causes systemic yellow foliar mosaic symptoms, while PepGMV-D strain (D) causes distortion of 1st–6th expanding leaves, and asymptomatic infection of subsequently developing leaves, like other known ‘recovery’ phenotypes. Infections established with DNA-A Mo and D components expressing red-shifted green fluorescent protein in place of coat protein and in situ hybridization, showed PepGMV-Mo localized to phloem and mesophyll cells, while -D was mesophyll restricted. Alignment of PepGMV-Mo and -D DNA-B components revealed three indels upstream of the BC1 gene that encodes the movement protein (MP). To determine if this non-coding region (*BC1) D-strain MP putative promoter contributed to ‘recovery’, plants were inoculated with chimeric DNA-B Mo/D components harboring reciprocally exchanged *BC1, and wild-type DNA-A Mo and D components. Symptoms were reminiscent but not identical to wild-type -Mo or -D infection, respectively, suggesting ‘recovery’ cannot be attributed solely to the *BC1. Both BC1 and D*BC1 were targeted by post-transcriptional gene silencing; however, ‘recovered’ leaves accumulated fewer transcripts and 21–24 nt vsiRNAs. Thus, inefficient in planta movement of PepGMV-D is associated with a non-pepper-adapted ‘defective’ BC1 that facilitates hyper-efficient PTGS, leading to BC1 transcript degradation that in turn limits virus spread, thereby recapitulating disease ‘tolerance’.

## 1. Introduction

Begomoviruses (genus, *Begomovirus*; family, *Geminiviridae*) [1] have a small, circular, single-stranded (ss) DNA genome, and infect many cultivated and uncultivated plant species (eudicots) in the tropics and subtropics. They are transmitted by the whitefly vector *Bemisia tabaci* (Genn.) cryptic species group [2,3]. Bipartite begomoviruses have two genome components, referred to as the DNA-A and DNA-B components, respectively. The DNA-A component has one open reading frame in the sense orientation that encodes the coat protein gene (*AV1* or *CP*) and four overlapping open reading frames (ORFs) on the complementary sense DNA (*AC1* or *Rep*; *AC2* or *Trap*; *AC3* or *REn* and *AC4*) that encode proteins involved in replication of the viral genome, regulation of viral gene expression, and suppression of plant host defense pathways [4,5,6]. The DNA-B component encodes two ORFs, one on the viral (*BV1*) and complementary (*BC1*) sense strands, respectively [7,8]. The BC1 or MP is required for viral DNA movement between the nucleus and cytoplasm [9,10], while specific interactions between the BC1/MP and BV1/NSP mediate cell-to-cell movement [10,11,12,13,14,15]. In addition, the BC1/MP interacts with a nuclear encoded 70 kDa chaperone at the cell periphery to mediate intercellular movement and chloroplast co-localization [16,17].

The bipartite *Pepper golden mosaic virus* (PepGMV) comprises a complex of three or more strains that infect pepper and tomato crops in Central America, Mexico, and the southwestern US, including Arizona and Texas [18,19,20,21,22]. The DNA-A and DNA-B component nucleotide sequences have been determined from infectious clones of PepGMV-Serrano (PepGMV-Ser), PepGMV-Mosaic (PepGMV-Mo), and PepGMV-Distortion (PepGMV-D), and the shared pairwise sequence identities among the three PepGMV components ranges from 91 to 96% for DNA-A and from 84 to 99% for DNA-B [23]. Anaheim pepper plants infected with PepGMV-Mo and PepGMV-Ser develop yellow–green foliar mosaic and golden foliar mosaic symptoms, respectively, and both are transmissible by the whitefly vector. Symptoms associated with PepGMV-D consist of a mild foliar mosaic and distortion that develop on the inoculated and subsequently developing 4–6 leaves, while subsequently developing leaves are symptom-free, and consistent with other previously reported symptom ‘recovery’ phenotypes [24]. Although PepGMV-D viral DNA is detectable post-inoculation (PI) in the newly developing leaves by polymerase chain reaction (PCR) amplification, the virus is not transmissible by the whitefly vector, except when in mixed infection with PepGMV-Mo. In contrast, PepGMV-Mo does not require PepGMV-D to infect pepper [23]. Infectivity studies with reciprocally reassorted DNA-A and –B genomic components, e.g., heterologous DNA-A and -B components of PepGMV-Mo, PepGMV-Ser, and -D strains have shown that all wild-type and genomically reassorted combinations of the PepGMV DNA-A and DNA-B components are infectious in pepper, and that pepper plants infected when inoculated with the PepGMV-D cognate components or with heterologous combinations with the B component of -D strain, exhibit a ‘recovery’ phenotype [23].

Under natural conditions, other begomovirus–host plant combinations are known to exhibit ‘recovery’ from disease symptoms, a response that is manifest as the remission of symptoms or dramatically reduced symptom severity [25,26,27]. Symptom recovery from a previously symptomatic infection has been associated with reduced virus accumulation resulting from gene silencing due either to post-transcriptional gene silencing (PTGS) and/or transcriptional gene silencing (TGS) plant host defense mechanisms [5,28,29]. The intensity of viral-induced post-transcriptional gene silencing (PTGS) can vary with different virus–host plant combinations [30]. In the PepGMV study system, infected plants exhibiting ‘recovery’ have also been associated with viral small interfering RNAs (vsiRNAs) accumulation in symptomatic and ‘recovered’ pepper leaves [31,32]. Further, silencing of PepGMV in infected pepper plants was shown to involve both post-transcriptional gene silencing (PTGS) and transcriptional gene silencing (TGS), acting together with silencing directed toward PepGMV coding and non-coding regions of the genome [32]. The role of siRNAs in the plant growth and development is well documented, as is the role of vsiRNAs in the establishment of tolerance to virus infection, or a response considered to represent a state between full-blown disease or susceptibility, and resistance [33,34]. Such host-virus interactions can readily lead to the accumulation of significant virus loads while resulting in minimal effects on plant growth, reproduction, and yield. In at least some instances, these effects have been attributed to reprogramming of gene expression and basal defense responses [33,35,36]. Thus, understanding the specific mechanisms involved in establishing a state of tolerance is expected to be instrumental in guiding the development of virus-resistant crops [34,37].

For certain begomovirus–host combinations, ‘tissue tropisms’ have been shown to involve phloem-restricted or phloem- and/or mesophyll-restricted invasion, where identified *cis*-acting viral element(s) located upstream of the BV1-coding region, and *trans*-acting functions contributed by viral AC2 itself, are involved in transcriptional regulation, including for TGMV [38] and *Cabbage leaf curl virus* [39] the regulation of NSP expression. Based on evidence that NSP—and virus coat protein (CP)—interact with cellular proteins [40,41,42], it has been proposed that AC2 binds dsDNA and that host factors act through a 50-nucleotide (nt) viral element to drive BV1 expression that in turn mediates viral movement [43]. Also, for certain bipartite begomoviruses, AC2 is essential for CP expression, by activation in mesophyll cells and de-repression of expression in vascular tissues [38,42,44]. Further, a bipartite 108 base pair (bp) regulatory element was shown to be essential for AC2-mediated activation of the BV1 promoter, indicating that AC2-mediated activation is a common regulatory circuit used by bipartite begomoviruses [45]. In general, long-distance movement and invasion of plant viruses adheres to the route used for photo-assimilate translocation [10,11,12,13,14]. Typically, viruses move through cellular barriers including the bundle sheath, vascular parenchyma, and companion cells and then are loaded into the distal sieve elements. Distal sieve elements unload viruses into companion cells and inversely cell to cell into bundle sheath and mesophyll cells.

Among the different PepGMV strains, analysis of the DNA-B component sequences revealed nucleotide polymorphisms that resulted in predicted amino acid changes within the non-coding region, located approximately 300-nt upstream from the start codon of the BC1 ORF [23]. Hereafter, this non-coding region of the MP is referred to as the *BC1 region. Although all four PepGMV strains are capable of infecting ‘Roma’ tomato, only the PepGMV-D strain does not cause full-blown, persistent symptoms in ‘Anaheim’ pepper, which suggests that it is poorly adapted to pepper. This probably explains why it has only been found in co-infection with another PepGMV strain, e.g., Mo strain [23]. Differences in the MP-putative promoter region sequences led to the hypothesis that differential BC1 expression might be associated with impeded cell-to-cell movement of viral DNA and that the BC1 non-coding region, upstream of the region encoding the viral MP required for cell-to-cell movement and NSP-coordinated functions, could be associated with the characteristically distinct symptom phenotypes that develop in ‘Anaheim’ pepper plants systemically infected with PepGMV-Mo and -D, respectively. The objective of this study was to probe the suspect role of the *BC1 non-coding region of PepGMV-Mo and -D strains in tissue tropism and the induction of PTGS/TGS that are herein hypothesized to lead to ‘host recovery’, exemplified by the susceptible and tolerant phenotypes observed in ‘Anaheim’ pepper plants infected with the Mo and D strains of PePGMV, respectively.

## 2. Materials and Methods

### 2.1. Virus Constructs

DNA restriction enzymes were obtained from Invitrogen (Carlsbad, CA, USA), and in vitro recombination and other DNA manipulations were carried out using standard molecular biology methods [46]. The sequences corresponding to the cloned PepGMV DNA-A and -B components, respectively, were previously deposited in the GenBank sequence database, as AY928512 and AY928513 for the -Mo strain, and AY928514 and AY928515 for the -D strain [23]. The unit-genome length DNA-A component for PepGMV-D and PepGMV-Mo were subcloned from previously constructed dimers cloned in the pGEM T-Easy plasmid vector (Promega, Madison, WI, USA), using *EcoR*I to produce viral monomeric components, referred to hereafter, as pDA and pMoA, respectively. The unit-length DNA-B component of PepGMV-D was subcloned into the pGEM-7zf (+) plasmid vector (Promega, Madison, WI, USA) as an *EcoR*I fragment, and the full-length DNA-B component of PepGMV-Mo was subcloned as a *Hind*III fragment, yielding ‘pDB’ and ‘pMoB’, respectively. The viral monomers were released from the respective dimers constructed previously [19,23] by restriction digestion, and served as the cloned monomeric components for subcloning of the BC1 ORF and putative promoter and the viral AV1 ORF region. The PCR amplicon sequences obtained downstream were verified by Sanger DNA sequencing prior to subcloning, and used to create the pDA, pMoA, pDB and pMoB constructs, each carrying a unit-length viral genomic (monomeric) component, respectively.

### 2.2. Confocal Microscopy and In Situ Hybridization of GFP PepGMoV-Mo and -D DNA Components

The cloned DNA-A components of PepGMV-Mo and -D were engineered to express red-shifted green fluorescent protein (GFP) [47] by replacing the respective viral CP gene with RS-GFP, to yield pMoA:GFP and pDA:GFP. The cloned monomers were each digested with *EcoR*I to release the insert, combined with their respective cognate DNA-B, and biolistically inoculated to ‘Anaheim’ pepper seedlings using the BioRad Biolistic Particle Delivery System PDS-1000/He or high-pressure helium gene gun (Hercules, CA, USA), as previously described [23]. The youngest, newly expanded leaves of pepper plants were removed from the inoculated seedlings 2–3 weeks post-inoculation and mounted in double-distilled water on a microscope slide with a cover slip. The GFP fluorescence was visualized using a BioRad 1024 confocal scanning head (Hercules, CA, USA) on a Nikon microscope, based on a previously published method [48].

The hypothesis that the -D strain might be defective for movement in the phloem/vascular cells of ‘Anaheim’ pepper was investigated by in situ hybridization using fluorescently labeled BC1 to localize -D and Mo (positive control) in pepper seedlings inoculated with the cloned viral genome of the respective strains. The probe was PCR-amplified from the PepGMV BC1 gene from pMoB and pDB, respectively, to incorporate the digoxigenin (DIG) label, according to the manufacturer’s protocol (Boehringer Mannheim). Wild-type -Mo and -D were localized by in situ hybridization with the respective homologous-BC1 probe, using previously established conditions for blocking, hybridization, post-hybridization washes, and detection [49]. Newly developing leaves, 2–3 weeks post-biolistic inoculation, were stained and visualized by light microscopy to analyze the subcellular localization of the PepGMV DNA-A component, using previously described methods [50].

### 2.3. Reciprocal Exchange of BC1 ORF and Putative Promoter Region

**Figure 1:**
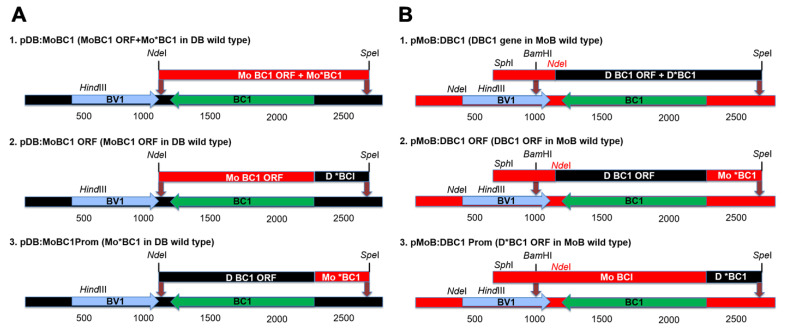
Schematic representation of the construction method used to build *Pepper golden mosaic virus* (PepGMV) DNA-B chimeras. (**A**) Subcloning *Nde*I/*Spe*I fragments (**top**) into distortion (D) strain DNA-B, black line (**bottom**) represents linearized wild-type distortion strain. (**1**–**3**) are the resultant chimeras constructed for -D strain. (**B**) Subcloning *Bam*HI/*Spe*I fragments (**top**) into mosaic (Mo) strain DNA-B, red line (**bottom**) represents linearized wild-type mosaic strain. (**1**–**3**) are the resultant chimeras for the Mo. The blue and green arrows represent the two ORFs on the DNA-B. The brown arrows point to the subcloning sites. *Nde*I site (in red) on the *Bam*HI/*Spe*I fragments was engineered because of the presence of another site on DNA-B Mo at the BV1 5′ end.

High fidelity PCR amplification using *Pfu* DNA polymerase (Strategene, La Jolla, CA, USA) was performed according to the manufacturer’s recommendations. Because *Pfu* is a proofreading polymerase and the resultant products have a blunt end, an ‘A’ overhang was added to the amplicons using *Taq* DNA polymerase prior to ligation and cloning into the pGEM T-Easy plasmid vector. For each construct, the fragment was amplified using the respective plasmid as template, with primers corresponding to the respective nt coordinates, as shown in Figure 1. Two unique restriction sites were engineered into each pDB amplicon to facilitate molecular cloning by replacing the 1292 bp fragment containing the BC1 and putative upstream promoter region, with the homologous fragment from pMoB, *Nde*I (nt coordinate 1147) and *Spe*I (nt coordinate 2438) sites in pDB (Figure 1A). The *Nde*I/*Spe*I fragment was subcloned into pDB using *Nde*I and *Spe*I to generate the pDB:MoBC1 clone (Figure 1(A1)). Using a similar strategy, the pDB:MoBC1 ORF, and pDB:MoBC1 Prom were constructed (Figure 1(A2,3)). Unlike pDB, the pMoB lacked an *Nde*I site at the 1147 nt coordinate; therefore, an *Nde*I site was engineered into the *Sph*I/*Spe*I 1704 bp fragment (located between nt coordinates 729 and 2432) using primers corresponding to the respective site (Figure 1B). The *Sph*I/*Spe*I fragment was cloned into the pGEM7zf (+) plasmid vector (Promega, Madison, WI, USA), and used to prepare constructs for subcloning. For the gene constructs, the *Sph*I/*Spe*I construct containing the -D BC1 ORF and the -D putative promoter, located between *Nde*I and *Spe*I sites, was subcloned into pMoB using *Bam*H1/*Spe*I, yielding pMo:DBC1 (Figure 1(B1)). Similarly, the pMo:DBC1 ORF and pMo:DBC1 Prom region constructs were engineered using an analogous strategy (Figure 1(B2,3)).

### 2.4. Replacement of the Coat Protein ORF with Soluble Modified Red Shifted GFP

The soluble-modified, red-shifted green florescent protein (smRS-GFP) [51] was amplified from the pJRT010 clone [52] using GFP (Nco-F) and GFP (Bs-R) primers. The amplified fragment was ligated to pMoA and pDA, as a *Bsm*I-*Nco*I fragment to produce pMoA:GFP and pDA:GFP, respectively. The start and stop codons of the coat protein coding region (AV1) were maintained in the smRS-GFP ORF. To circumvent an unwanted internal *Bsm*I site, the complete smRS-GFP ORF was subcloned by PCR-amplification of a fragment digested with *Nco*I, and partially digested with *Bsm*I, prior to subcloning into the full-length DNA-A component. The accuracy, i.e., fidelity of PCR amplification and restriction digestions were verified by confirmatory DNA (Sanger) sequencing and primer walking at The University of Arizona Genomics and Technology Center (GATC), Tucson, AZ, USA.

### 2.5. Biolistic Inoculation of PepGMV Clones

The two wild-type strains and the respective chimeras were biolistically inoculated into ‘Anaheim’ pepper and *N. benthamiana* seedlings (3–4-leaf stage). Inoculated plants were maintained in a growth room at 24 °C constant temperature and 12:12 h light/dark cycle. Plants were observed daily, and symptom development and phenotype were carefully documented for homologous and heterologous DNA-A and -B component chimeric and wild-type combinations. ‘Anaheim’ pepper leaves mock inoculated with buffer, minus the cloned viral genomes, and non-inoculated pepper plants were included as negative experimental controls, as previously described [23].

### 2.6. Viral DNA and RNA Accumulation in ‘Anaheim’ Pepper Leaves Infected with Wild-Type and Chimeric Infectious Clones

For Southern blot analysis, approximately 5 µg of total DNA from leaves 1 to 6 above the inoculated point was loaded in 1% agarose gels. Gels were capillary transferred to a ‘Hybond’ nitrocellulose membrane (positively charged) according to the manufacturer’s instructions. Membranes were UV crosslinked (Stratagene, La Jolla, CA, USA) and stored at 4 °C. Hybridization was carried out overnight at 50 °C using pre-hybridization and hybridization protocols, according to Hutvagner et al., 2000 [53]. The signal was scanned after carrying out a 16 h exposure with a Storm phosphor-imager. After hybridization, the probe was removed from the membrane by incubation in 0.1x SSC and 0.5% SDS solution, heated to boiling and added to pouches containing the membranes, for a 15 min incubation, and the process was repeated a second time [54]. The PepGMV-Mo DNA-A component was labeled using [α-^32^P] CTP with the Redi Prime II kit (GE HealthCare, Chicago, IL, USA), according to the manufacturer’s protocol.

For Northern blot analysis, leaves were collected from 5 to 8 plants, pooled, and processed. The samples collected were as follows: (a) PepGMV-Mo leaves 1 and 2 symptomatic; (b) PepGMV-Mo leaves 5 and 6 symptomatic; (c) PepGMV-D leaves 1 and 2 symptomatic; (d) PepGMV-D leaves 5 and 6 “recovered”; (e) PepGMV-Mo A with pMoB:DORFProm leaves 1 and 2 ‘mild symptoms’; (f) PepGMV-Mo A with pMoB:DORFProm leaves 5 and 6 ‘recovery symptoms’; (g) PepGMV-D A component with pDB:MoORFProm leaves 1 and 2 wild-type symptoms; (h) PepGMV-D B component with pDB:MoORFProm leaves 5 and 6 wild-type symptoms; and (i) virus-free pepper plants. Approximately 5 g of leaf tissue was used for total RNA isolation using TRIZOL (Life Technologies, Waltham, MA, USA), according to the manufacturer’s instructions. The RNA pellets were air-dried, dissolved in 500 μL of nuclease-free water, and stored at −80 °C. One microliter of each sample was quantified by nanodrop spectrophotometry. For the northern blots, 20 μg total RNA was loaded onto the gel, and gels and blots were processed using the NorthernMax-Gly Glyoxal-Based System for Northern Blots (Life Technologies, Carlsbad, CA, USA), according to the manufacturer’s instructions.

The BC1 and 18S rRNA gene probes were used as internal hybridization controls. Probes were PCR-amplified with the following primers: ~700 bp product of the BC1 gene of -D and -Mo strains, and ~300 bp of the 18S gene: MP-F5′-TAGTCCTTCAGTTTCCTTCCAC-3′ and MPR-5′-ATTGGGCCATTGACATTGTT-3′; 18Spepper-F5′-GCGGAAGGATCATTGTTGAA-3′ and 18Spepper-R5′-GAGAGCCGAGATATCCGTTG-3′. The probes for detecting BC1 transcripts of each respective begomovirus strain were prepared by PCR-amplification with RedTaq DNA polymerase (Sigma-Aldrich, St. Louis, MO, USA) using the following conditions: 94 °C for 5 min, followed by 30 cycles of 94 °C for 1 min, 55 °C for 1 min and 72 °C for 1 min. PCR products were fractionated by agarose gel (1%) electrophoresis and gel eluted. One microliter of each probe was labeled using the Amersham Rediprime^TM^ II DNA Labeling System (GE Healthcare Life Sciences) following the manufacturer’s instructions. Probe (1 μL) was mixed with 44 μL of TE, denatured at 95 °C for 10 min, and immediately cooled on ice for 5–10 min. An aliquot of 45 μL was added to one tube of RediPrime. For labeling, 5 μL of αP^32^ dCTP 50 μCi (Perkin Elmer catalog number BLU513H) was used per reaction, and added to the RediPrime/Probe mixture and incubated at 37 °C for 1 h. After incubation the probes were denatured at 95 °C for 10 min and snap cooled on ice, prior to their addition to the hybridization reaction in buffer. Hybridization reactions were carried out according to the manufacturer’s protocol, NorthernMax-Gly Glyoxal-System for Northern Blots (Abion, Foster City, CA, USA). The membranes were exposed using a phosphor screen (GE HealthCare, Chicago, IL, USA) and scanned with a Storm 860 molecular imager (Amersham Biosciences Corp, Piscataway, NJ, USA).

### 2.7. Small RNA Analysis

Total RNA was purified from pooled liquid nitrogen-powdered leaves (10/plant) collected from test plants and prepared as described above, using TRIzol^TM^ Reagent (Thermo Fisher Scientific, Grand Island, NY, USA), according to the manufacturer’s instructions. The low molecular weight (LMW) RNA was isolated from total RNA, as described previously [32]. The concentration of LMW RNA was determined using a NanoDrop spectrophotometer (Thermo Fisher Scientific, Grand Island, NY, USA) and 15 μg of the sample was re-dissolved in 15 μL of loading buffer, denatured by heating to 65 °C for 5 min, and fractionated on a 17% polyacrylamide 8 M urea denaturing gel. The microRNA Marker (New England Biolabs, Ipswich, Massachusetts, USA) consisted of single-stranded RNA oligonucleotides of 17, 21 and 25 bases, respectively.

Electrophoresis was carried out in a Protean Apparatus (BioRad, Hercules, CA, USA) at 150 V for 2 h, and gels were stained with SYBR-Gold. The RNA was transferred in 0.5x TBE buffer to a Nylon Nytran Supercharged membrane (Whatman, Maidstone, UK) at 10 V for 1-h, followed by UV crosslinking (Stratagene, La Jolla, CA, USA). Membranes were probed with a 700 bp fragment of the BC1 gene specific for the respective PepGMV strain. The loading control was determined by visualization of the ethidium bromide-stained gels. Probes were labeled using [α-^32^P] CTP using the Redi Prime II kit (GE HealthCare Chicago, IL, USA). Hybridization was carried out overnight at 42 °C, according to Hutvagner et al., 2000 [53]. Gels were scanned 3-d post-initial exposure using a Storm Phosphor-imager.

## 3. Results

### 3.1. Symptom Phenotype in Pepper Inoculated with PepGMV Wild-Type and Chimeric Viruses

Pepper seedlings were inoculated with different combinations of the six DNA-B component mutants (Table 1) and the DNA-A component of the two virus strains, respectively. These results demonstrated that swapping either the putative promoter region or the BC1 ORF resulted in the development of a somewhat unique symptom phenotype, respectively, compared to wild-type symptoms associated with the homologous DNA-A and -B components of -Mo and -D, respectively (Table 1).

**Figure 2:**
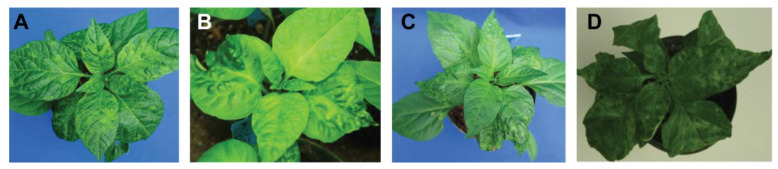
Characteristic symptoms in inoculated ‘Anaheim’ pepper plants. (**A**) PepGMV-Mo wild-type inoculated ‘Anaheim’ pepper plant at 15 dpi; (**B**) PepGMV-Mo strain DNA-A and DNA-B background containing pMoB:DBC1 Prom inoculated pepper plant at 15 dpi; (**C**) PepGMV-D wild-type inoculated pepper plant at 15 dpi; (**D**) PepGMV-D strain DNA-A and DNA-B background containing pDB:MoBC1 Prom inoculated pepper plant at 15 dpi.

To determine whether the *BC1 region was involved in symptom phenotype development, infectious clones of the two PepGMV wild-type and chimeric viruses were inoculated to ‘Anaheim’ pepper plants (Figure 2). Results indicated that the chimeric component harboring the *BC1 region from PepGMV-Mo DNA-B, when co-inoculated with DNA-A -D (Figure 2D) produced mosaic symptoms in pepper seedlings and pepper seedlings did not exhibit the recovery phenotype characteristic of plants infected by the homologous PepGMV-Mo A and B components (Figure 2A). In contrast, co-inoculation of pepper plants with the DNA-B component chimera containing the *BC1 of the PepGMV-D BC1 gene and the DNA-A -Mo resulted in development of mosaic symptoms, and a ‘modified recovery’ phenotype (Figure 2B) that was less severe than the wild-type distortion symptom characteristic of plants infected by the homologous PepGMV-D A and B components (Figure 2C).

Pepper plants inoculated with wild-type PepGMV-Mo developed chlorotic spots and yellow mosaics at 7 days post-inoculation (dpi), and symptoms continue developing throughout from leaves 1 to 6 above the inoculated point (Figure 2A). When pepper seedlings were inoculated with PepGMV-Mo DNA-A wild-type and pMoB:DBC1Prom, a mild phenotype was observed in the first 1–2 leaves above the point of inoculation, followed by a recovery phenotype in the newly developing leaves (Figure 2B). Pepper plants inoculated with the wild-type PepGMV-D strain developed foliar chlorosis and yellow mosaic symptoms in the inoculated and first newly developing leaves 7 dpi (leaves 1–4). At 15 dpi, definitive ‘recovery’ symptoms, or symptom-free leaves were observed following inoculation, e.g., on leaves 5 and 6 (Figure 2C). When pepper seedlings were inoculated with PepGMV-D DNA-A wild-type and pDB:MoBC1 Prom, yellow mosaic symptoms appeared on leaves 1–6 that developed from above the point of inoculation, and no ‘recovery’ was observed in upper leaves (Figure 2D), as was typical for pepper plants inoculated with wild-type PepGMV-Mo (Figure 2A). Thus, pepper plants (Figure 2D) did not develop recovery symptoms similar to those observed in plants inoculated with wild-type PepGMV-D (Figure 2C). Results indicated that the putative promoter region, upstream of *BC1 coding region of Mo, yielded a near-phenocopy of the symptoms characteristically exhibited by ‘Anaheim’ pepper plants infected with wild-type PepGMV-Mo. These results indicated that pepper plants systemically infected with the reciprocal chimeric *BC1-Mo, or -D cloned genomes, developed wild-type persistent yellow mosaic and ‘recovery’ symptoms, respectively, while the positive control plants infected with homologous or cognate -Mo or -D DNA-A and -B components exhibited strain-specific symptoms.

### 3.2. Virus Accumulation in ‘Anaheim’ Plants Infected by Wild-Type PepGMV-Mo and -D Strains

**Figure 3:**
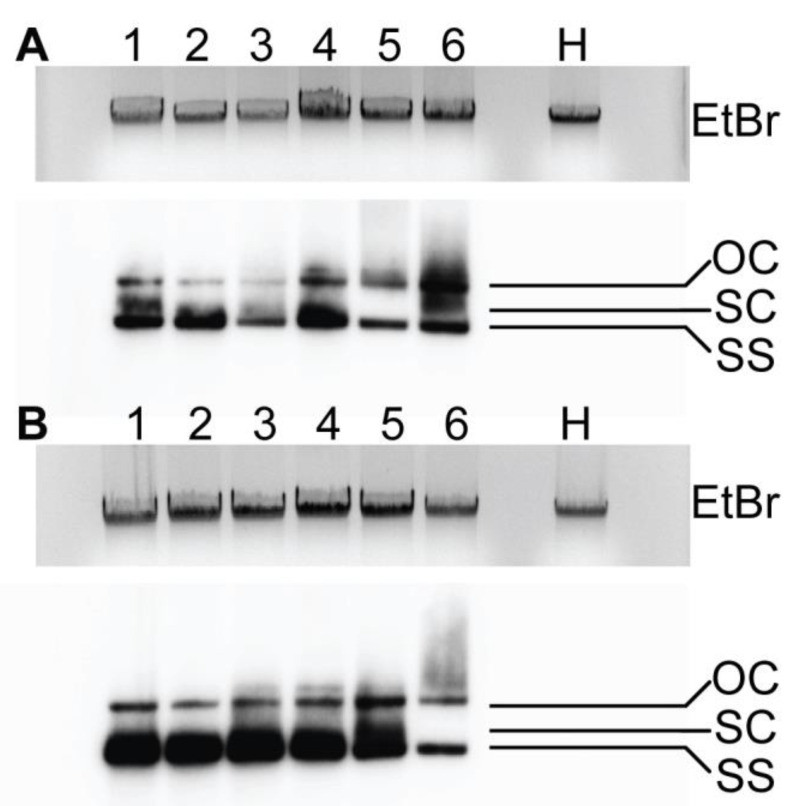
Viral DNA accumulation in PepGMV-Mo and PepGMV-D wild-type strains. Total DNA was fractionated by 1% agarose gel electrophoresis blotted onto nitrocellulose membranes and hybridized with PepGMV-Mo as probe. Approximately 5 mg of total DNA were loaded in each lane. (**A**) PepGMV-Mo strain leaves 1 to 6 and H = virus-free or ‘health’ control; (**B**) PepGMV-D strain leaves 1 to 6 and H = healthy or virus-free control. OC = open circle, SC = super coiled, and SS = single stranded.

To determine if PepGMV -Mo and -D wild-type viral DNA accumulation was directly associated with symptom phenotype, total DNA was isolated from leaves 1–6 that developed post-inoculation, transferred to a plus-charged nylon membrane using capillary transfer [47], and hybridized using the cloned PepGMV-Mo DNA-A component as probe. Results demonstrated that viral DNA was detected in all the leaves, regardless of whether they were symptomatic or displayed the recovered phenotype (Figure 3). In PepGMV Mo-inoculated plants, relatively similar levels of viral DNA accumulation were observed in the symptomatic leaves 1 through 6, all of which exhibited wild-type disease symptoms, and additionally, an increase in relative DNA accumulation was observed in leaf 6 (Figure 3A). In PepGMV-D-inoculated plants, the relative viral DNA accumulation was high and consistent for leaves 1 through 5, whereas a dramatic decrease in relative accumulation was documented in leaf 6, which subsequently displayed the ‘fully recovered’ phenotype, with no detectable symptoms (Figure 3B). Symptoms observed in PepGMV-Mo-infected plants showed similar levels of relative viral accumulation in all leaves, whereas, for PepGMV-D-infected plants that exhibited the recovery phenotype, there was an inverse correlation between relative levels of viral DNA accumulation.

### 3.3. Localization of the PepGMV Viral Genome in Pepper Seedlings

**Figure 4:**
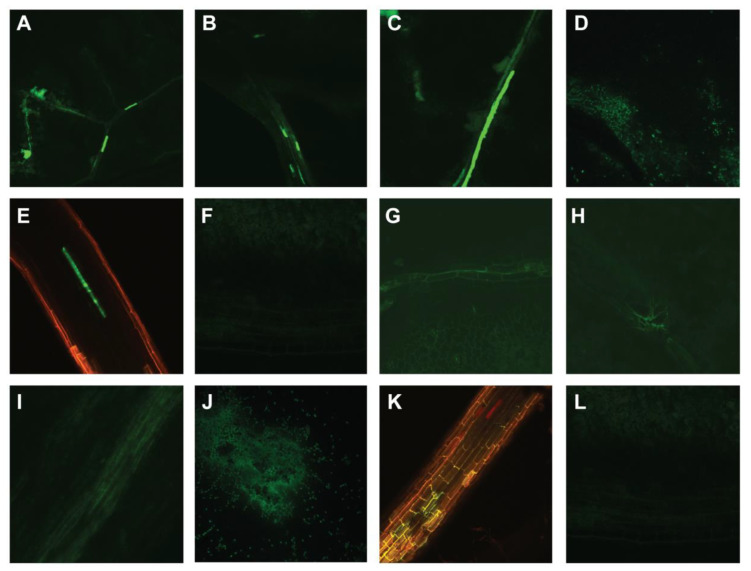
Fluorescence microscope images showing *Pepper golden mosaic virus* (PepGMV) wild-type strains (Mo and D) distribution along transverse sections of leaves and roots of pepper infected plants. Figures (**A**–**F**) correspond to PepGMV-GFP detection in Mo-inoculated pepper plants. (**A**) leaf 1; (**B**) leaf 3; (**C**) leaf 5; (**D**) inoculated leaf; (**E**) root; (**F**) mock inoculated. Figures (**G**–**L**) correspond to PepGMV-GFP detection in D-inoculated pepper plants. (**G**) leaf 1; (**H**) leaf 3; (**I**) leaf 5; (**J**) inoculated leaf; (**K**) root; (**L**) mock inoculated.

When wild-type PepGMV-Mo and -D strains were localized in pepper leaves using GFP expression (as coat protein replacements and DNA-A components) and tracked using confocal imaging to monitor viral movement, the GFP fluorescence revealed a robust signal indicating PepGMV-Mo presence, while an extremely faint signal was observed in PepGMV-D-infected plants, respectively. For the PepGMV-Mo-infected pepper seedlings fluorescence was visible in symptomatic tissues of both the inoculated and newly expanding (post-inoculation) leaves, and viral presence was documented in the phloem-adjacent cells and tissues (Figure 4A–D) and in roots (Figure 4E). In contrast, the mock-inoculated pepper seedlings showed no fluorescence indicating absence of virus (Figure 4F).

Alternatively, the faint PepGMV-D GFP-associated fluorescence was observed primarily in phloem of symptomatic pepper leaves (Figure 4G), and signal was barely discernable or not detectable at all in leaves that either exhibited mild symptoms or were asymptomatic (Figure 4H,I). Also, fluorescence was observed in the inoculated leaves (Figure 4J) and in pepper plant roots, indicative of virus presence (Figure 4K).

**Figure 5:**
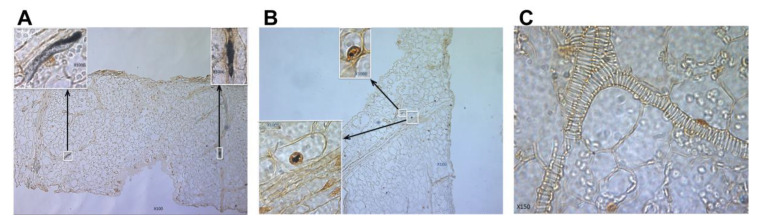
In situ localization of *Pepper golden mosaic virus* (PepGMV) in pepper plants inoculated with (**A**) mosaic strain: (**B**) distortion strain and (**C**) mock-inoculated, negative control. Pepper plants exhibiting PepGMV-Mo and -D strain-specific symptoms (**A**,**B**) and no symptoms (**C**) were sectioned and hybridized with BC1:Mo- or BC1:D-specific DIG-labeled probes and viewed at the ×100 and ×1000 magnification. Inserts represent ×1000 of boxed areas.

The extent of virus localization was further analyzed in situ hybridization experiments with leaves detached from pepper plants inoculated as described above. The in situ hybridization results were highly consistent with the observations observed by confocal imaging, confirming that PepGMV-Mo was localized to the phloem and adjacent cells and tissues in symptomatic leaves (Figure 5A), whereas PepGMV-D appeared to have been capable of movement at least to some extent, into the recovered leaf (Figure 4I). Even so, the fluorescent signal was relatively weak, and movement appeared possibly to involve cells adjacent to the phloem, instead of the phloem itself (Figure 5B), suggesting phloem-loading may have been impaired. Thus, although both strains were able to move into the newly developed leaves, there was a striking difference in the intensity of the fluorescence and the hybridization signals, respectively, suggesting lower accumulation of PepGMV-D in leaves, compared to the more robust accumulation of PepGMV-Mo. These results indicated that PepGMV-Mo systemic (long-distance) movement occurred by way of the ‘anticipated’ route of phloem assimilate translocation, or through phloem and the adjacent cells, whereas the movement-defective PepGMV-D spread was detectable in only the adjacent cells in ‘recovered’ leaves. These observations may in part explain why PepGMV-D can be detected by PCR amplification in the asymptomatic ‘recovered’ leaves, while at the same time, it is not whitefly transmissible [22]. A hypothesis relevant to the latter observation is that PepGMV-D DNA does not efficiently access the terminally differentiated cells, the site in the plant host where replication is expected to occur. If so, replication occurs at extremely low levels, in turn, leading to reduced translation of viral proteins, in this scenario, the coat protein, which is essential for ssDNA encapsidation and virion production. Another possibility could be that replication, translation, and virion assembly occur, but that virion accumulation is so minimal that ingestion of virions by the whitefly vector is below the threshold required for vector-mediated acquisition, precluding virus transmission. Either hypothesis can explain another key observation that PepGMV-D is only transmitted by the whitefly when it occurs in mixed infection with PepGMV-Mo or other competent strains of PepGMV, effectively, rescuing the movement-impaired PepGMV-D strain, to effect vector-mediated transmission between ‘Anaheim’ pepper plants. Such an impediment has not been observed for PepGMV-D infections of tomato plants, in which the D-strain is capable of causing full-blown systemic infection and is whitefly transmissible on its own, potentially indicative of the involvement of pepper-specific factor(s) that confer or are associated with ‘tolerance’ in ‘Anaheim’ pepper to PepGMV-D, and that when identified, will be of great interest.

Finally, the inability of PepGMV-D to incite full-blown systemic infection in tomato but not in pepper, suggests that PepGMV-D likely evolved initially as a tomato-infecting begomovirus, and post-emergence in pepper crops has undergone only the first steps required to complete the successful ‘host shift’ to pepper. This unique study system offers an opportunity to probe mechanisms in play, in this ‘host-shift’ scenario, with a plant host species/cultivar that is tolerant to PepGMV-D infection. This is despite its’ susceptibility to the other known PepGMV strains, which are apparently well adapted to ‘Anaheim’ pepper, and have not acquired detrimental mutations in the DNA-B component upstream putative promoter region, as documented for PepGMV-D.

### 3.4. Transcript Accumulation Attributed to PepGMV BC1 Gene Expression Based on Northern Blot Analysis of Inoculated ‘Anaheim’ Pepper Plants

**Figure 6:**
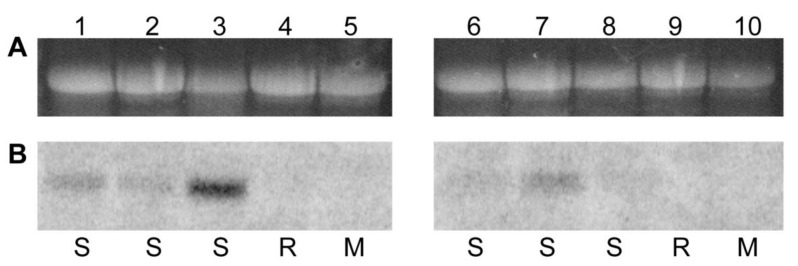
Northern blot hybridization of total RNA isolated from pepper plants inoculated with *Pepper golden mosaic virus* (PepGMV) wild-type and chimeric viruses. (**A**) Loading control (EtBr stain). Approximately 20 mg of total RNA were loaded in each lane. (**B**) Blots hybridized with 700 nt of BC1 gene as probe. S = symptomatic; R = recovered. Lanes: (1) PepGMV-Mo leaves 1 and 2 symptomatic; (2) PepGMV-Mo leaves 5 and 6 symptomatic; (3) PepGMV-D Leaves 1 and 2 symptomatic; (4) PepGMV-D Leaves 5 and 6 recovered; (5) healthy pepper; (6) PepGMV-Mo with DNA-A and DNA-B background containing pMoB:DBC1 Prom Leaves 1 and 2 mild symptoms; (7) PepGMV-Mo with DNA-A and DNA-B background containing pMoB:DBC1 Prom Leaves 5 and 6 recovered; (8) PepGMV-D with DNA-A and DNA-B background containing pDB:MoBC1 Prom Leaves 1 and 2 symptomatic; (9) PepGMV-D with DNA-A and DNA-B background containing pDB:MoBC1 Prom Leaves 5 and 6 symptomatic; (10) Healthy pepper.

To evaluate if ‘slowed’ cell-to-cell movement could be associated with the ‘recovery’ phenotype, and/or reduced expression of BC1 transcripts, transcript levels were quantified by northern blot analysis using the BC1 gene as probe. Transcripts were detectable in all the symptomatic pepper leaves inoculated with PepGMV-Mo. In contrast, virus transcripts were undetectable in leaves of all the analogous developmental stage(s) of plants infected with wild-type PepGMV-D BC1, which exhibited the ‘recovery’ phenotype (Figure 6).

To investigate the suspect involvement of the putative BC1 promoter region in the genesis of the ‘recovery’ phenotype, pepper seedlings were inoculated with the respective *BC1 -Mo and -D chimeric viruses harboring the reciprocal PepGMV-Mo and -D promoter region sequence. The accumulation of BC1 transcripts in pepper seedlings inoculated with PepGMV-Mo (-D promoter region) increased in ‘recovered’ leaves, compared to the chimeric PepGMV-D (-Mo putative promoter region)-inoculated plants, in which symptomatic leaves showed no detectable levels of BC1 transcripts (Figure 6).

### 3.5. Small RNAs Homologous to the BC1 Gene: Accumulation in ‘Anaheim’ Plants Exhibiting Wild-Type and ‘Recovery’ Phenotype

**Figure 7:**
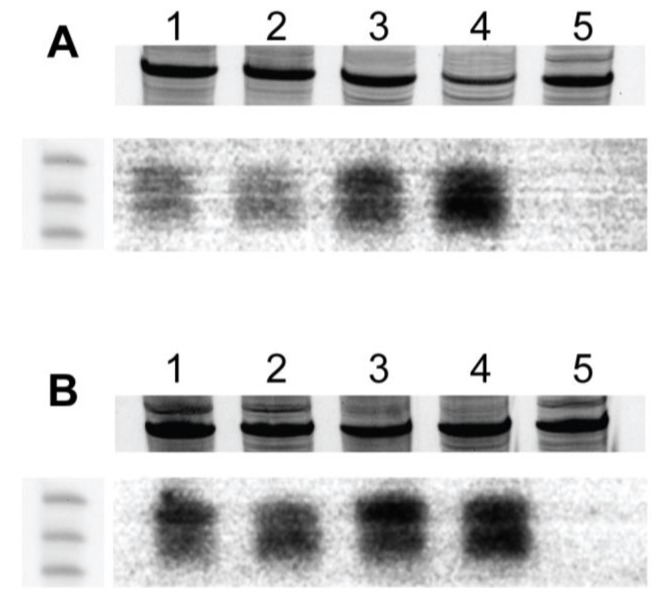
Northern blot analysis of vsiRNAs from pepper plants inoculated with each PepGMV-Mo and PepGMV-D wild-type strains and chimeras. The low molecular weight (LMW) RNA was fractionated by denaturing polyacrylamide gel electrophoresis and hybridized with the 700 bp probe from the BC1 gene (Movement protein gene). 15 mg of LMW RNA was loaded into each lane. (**A**) Lane (1) PepGMV-Mo strain symptomatic leaves 1 and 2; Lane (2); PepGMV-Mo strain symptomatic leaves 5 and 6; Lane (3) PepGMV-D strain symptomatic leaves 1 and 2; Lane (4) PepGMV-D strain recovered leaves 5 and 6: Lane (5); Mock inoculated control. (**B**) Lane (1) PepGMV-Mo with DNA-A and DNA-B background containing pMoB:DBC1 Prom mild symptomatic leaves 1 and 2; Lane (2) PepGMV-Mo with DNA-A and DNA-B containing pMoB:DBC1 Prom-recovered leaves 5 and 6; Lane (3) PepGMV-D with DNA-A and DNA-B containing pDB:MoBC1 Prom-symptomatic leaves 1 and 2; Lane (4) PepGMV-D with DNA-A and DNA-B containing pDB:MoBC1 Prom-symptomatic leaves 5 and 6; Lane (5) Mock inoculated control.

Small RNAs of 21–24 nt in size with sequence homology to BC1 were present in symptomatic and ‘recovered’ leaf tissue of pepper plants inoculated with wild-type PepGMV-D and PepGMV-Mo, respectively (Figure 7A). A comparison of small RNAs (21–24 nt) in leaves of pepper plants inoculated with wild-type PepGMV-Mo and -D showed that small RNA accumulation was greatest in the PepGMV-D ‘symptomatic’ and ‘recovered’ leaves, compared to significantly lower accumulation in PepGMV-Mo symptomatic leaves (Figure 7A). In fact, a slight increase in small RNA accumulation was observed in ‘recovered’ leaves of plants inoculated with PepGMV-D, compared to analogous leaves of PepGMV-Mo-infected plants (Figure 7A). The intensity of silencing associated with BC1 expression indicated that the pepper plants which had ‘recovered’ from PepGMV-D infection accumulated slightly lower levels of siRNAs, indicating greater BC1 transcript degradation, compared to PepGMV-Mo-infected plants.

Further, both PepGMV-chimeric virus-infected pepper plants accumulated 21–24 nt siRNAs, which are expected to share 100% sequence homology with their respective BC1 gene (Figure 7B). However, the symptomatic leaves of plants infected by PepGMV-Mo with DNA-A and DNA-B containing pMoB:DBC1 Prom chimeric virus accumulated the greatest amounts of 21–24 nt siRNAs (Figure 7B). No detectable siRNAs were documented in the non-inoculated, control pepper plants (Figure 7). Finally, the siRNA–host profiles showed that all ‘recovered’ PepGMV-D pepper plants had signatures of both TGS and PTGS silencing.

## 4. Discussion

PepGMV emerged as a begomoviral pathogen of tomato and/or pepper crops in Mexico and the southern U.S. during the mid-1990s. Multiple PepGMV strains were identified nearly simultaneously that shared high nucleotide sequence identity and yet caused different symptom in ‘Anaheim’ pepper plants, compared to the indistinguishable leaf curling symptoms in infected tomato plants. The PepGMV-Mo was found to cause bright golden foliar mosaic symptoms in ‘Anaheim’ pepper. In contrast, PepGMV-D, following its establishment in pepper plants by mechanical inoculation from an apparently mixed infection with PepGMV-Mo, was found to cause initial mild foliar mosaic symptoms, followed by progressively mild leaf ‘distortion’, and ultimately ‘recovery’ of plants, manifest as the absence of symptoms in the newest expanding leaves 12–14 days post-inoculation [26]. The genome of these closely related PepGMV strains, like other bipartite begomoviruses, encode two genomic components, referred to as DNA-A and DNA-B. All reciprocal combinations of the cloned infectious DNA-A and DNA-B components are capable of systemically infecting ‘Anaheim’ pepper plants, and the respective divergent DNA-B components have been associated with strain-specific symptom phenotypes [23].

In this study, ‘Anaheim’ pepper plants inoculated with the wild-type PepGMV-D or PepGMV-Mo DNA-A and –B components, respectively, when localized in planta by GFP-confocal microscopy and in situ hybridization, showed distinctively different patterns of association or capacity for ‘movement’ in the vascular tissues of pepper plants. Specifically, movement of PepGMV-D was impaired in the vasculature of the inoculated and subsequently developing leaves, with virus restricted to the mesophyll cells, while PepGMV-Mo was observed in both mesophyll and phloem cells (Figure 4 and Figure 5). This is consistent with the previously described symptom remission in pepper plants that resulted in a symptom phenotype reminiscent of disease ‘tolerance’ observed for PepGMV-D-infected plants, and less-than-wild-type (Mo) accumulation of -D strain in pepper leaves that develop after the initial infection in inoculated and approximately the first six expanding leaves, post-inoculation. In contrast, PepGMV-Mo was highly mobile in leaves and capable of full-blown, systemic infection of pepper plants which developed characteristic golden mosaic symptoms in all leaves of infected plants and was reminiscent of symptoms observed in field-infected pepper plants. Southern blot analysis of infected leaf tissue revealed significantly lower DNA accumulation in PepGMV-D ‘recovered’ leaf tissue, compared to all other leaf positions tested. In contrast, all leaves of pepper plants inoculated with PepGMV-Mo accumulated ‘wild-type’ or stable levels of detectable virus DNA and showed no discernable changes in -Mo accumulation. These results are consistent with the results of previous studies that reported a positive correlation between viral DNA accumulation and symptom severity [25,30,31,55]. The results presented here further support the hypothesis that impaired viral movement in phloem of ‘Anaheim’ pepper plants lead to gradually reduced viral DNA accumulation in leaves that develop above the point of PepGMV-D inoculation, leading to ‘recovery’ or a tolerant phenotype in infected pepper plants. For bipartite begomoviruses, the BC1 and BV1 genes are responsible for intra- and intercellular movement of viral DNA, respectively [10], and both are encoded on the DNA-B component, and therefore, one or both could feasibly contribute to the strain-specific differences in symptom phenotype previously reported in ‘Anaheim’ pepper plants infected with the PepGMV-Mo and -D strains [19,23].

The BC1 gene, or movement protein (MP), mediates virus cell-to-cell movement and long-distance movement through cooperative interaction with the nuclear shuttle protein BV1 [17]. The BC1 protein has been previously associated with distinct symptom phenotypes in virus-plant interactions [56]. Northern blot analysis showed higher levels of BC1 transcripts in leaves that developed post-PepGMV-Mo inoculation compared to those that developed post-PepGMV-D inoculation (Figure 6A), indicating that the -Mo BC1 protein is fully viable and capable of wild-type, cell-to-cell movement in ‘Anaheim’ pepper plants. Sequence alignments of PepGMV-Mo and -D BC1 coding regions revealed no apparent, predicted differences that could be attributable to differences such as those observed in cell-to-cell movement by either MP or to differential viral loads reported in infected pepper plants. However, inspection of the sequences upstream of -Mo and -D BC1 coding regions revealed a divergent region of ~300 nt in length located 5′-prime of the BC1 coding region. In other begomoviruses, coding regions and/or non-coding sequences upstream of the BV1 gene or BC1 gene identified as promoter regions, have been associated with specific symptom phenotypes and tissue tropisms [39,57]. These observations provide support for the involvement of putative promoter regions of less-well-studied begomoviruses in the development of distinct phenotypes, such as those observed in PepGMV-Mo- and PepGMV-D-infected ‘Anaheim’ pepper plants. The reciprocal exchange of the putative promoter regions (Figure 1) showed that strain-specific symptoms were reproducible for plants inoculated with chimeric virus harboring a 300 nt fragment of the putative MP promoter (*BC1). Mild symptoms, followed by ‘host recovery’, or a disease-tolerant phenotype, were observed in pepper plants inoculated with the -Mo chimera harboring the -D strain promoter, while plants inoculated with -D strain BC1 harboring the -Mo promoter, developed persistent golden mosaic symptoms and showed no evidence of tolerance to infection by the PepGMV-Mo strain (Figure 2).

Geminiviruses are known to be capable of inducing and suppressing plant host post-transcriptional gene silencing (PTGS) [28,58]. In this and previously published studies designed to investigate PepGMV-‘Anaheim’ pepper interactions, both viral coding and non-coding regions of PepGMV harbor small RNA targets. The BC1 gene and its non-coding upstream (putative) promoter region have been shown to be highly targeted for degradation, leading to an accumulation of homologous small RNAs [32]. Irrespective of PepGMV strain or chimeric virus, the BC1 gene is silenced by homologous small RNAs, which would be expected to result in fewer MP transcripts and therefore less viral MP. In this scenario, abated cell-to-cell viral movement and systemic infection appear to be likely. This hypothesis is supported by previous studies of pepper host-PepGMV interactions, albeit involving heterologous (non-cognate) viral components [21], which have demonstrated that viral-derived siRNAs accumulated to different levels in symptomatic and ‘recovered’ leaves [31,32]. Similar but different results are reported here for the homologous, cognate components as well as chimeric-MP constructs of PepGMV-Mo and PepGMV-D (Figure 7). Experiments further showed that although ‘Anaheim’ pepper plants inoculated with the respective wild-type DNA-A and chimeric DNA-B component, exhibited symptoms similar to but did not identically phenocopy wild-type symptoms, suggestive of greater than anticipated complexity in PepGMV-Mo and -D MP promoter region–host interactions.

The availability of the ‘Anaheim’ pepper-PepGMV study system to investigate contrasting susceptibility-tolerance phenotypes has advanced knowledge about this pathosystem and has demonstrated that recovery, and therefore disease tolerance, to PepGMV-D strain is associated in part with the differential accumulation of small RNAs via PTGS of PepGMV-Mo and -D BC1 sequences. Specifically, wild-type -Mo and -D strains and reciprocally exchanged BC1 promoter/ORF chimeras were differentially targeted by the host gene silencing pathway. And inoculation of ‘Anaheim’ pepper plants with cognate and chimeric-cognate PepGMV components yielded wild-type and similar ‘recovery’ phenotype symptoms, respectively. Collectively, these observations are consistent with the robust targeting of BC1 transcripts by both the PTGS and TGS host plant gene silencing pathway, respectively. Finally, the reduced accumulation of -D MP compared to -Mo MP transcripts that resulted from the differential host gene silencing is posited to lead to restricted cell-to-cell and systemic movement of the -D strain in ‘Anaheim’ pepper plants, increasing the susceptibility of the MP gene to silencing, and in turn, to less efficient replication over time due to its’ inability to access uninfected plant cells, compared to the unimpeded PepGMV-Mo host interactions.

Finally, these observations implicate potentially complex interactions involving the putative promoter region of the PepGMV-D MP which appears to be defective for competent interaction(s) with one or more putative transcription factors in ‘Anaheim’ pepper plants, potentially revealing a ‘hot spot’ to guide host transcription factor modification of pepper by gene editing, for example, to yield broad resistance to multiple pepper-infecting begomoviruses. Although still speculative, potentially, the binding of ‘Anaheim’-pepper-specific transcription factors to strain-specific, begomoviral *cis*-acting elements would be expected to differentially influence RNA polymerase II activity and its binding to sequences near the transcription start site required to initiate mRNA synthesis [33,35,36,59,60]. Studies to investigate the prospective differences in putative transcription factor(s) and their interactions with wild-type -Mo and mutant -D virus promoter regions in differentially susceptible host combinations tomato (susceptible: susceptible) and ‘Anaheim’ pepper (susceptible: recovery) are expected to advance the understanding of how plant hosts respond differentially, with reference to the co-evolution of the ongoing ‘arms race’ between host plant defenses and begomoviral invasion.

## Figures and Tables

**Table 1:** Results of biolistic inoculations of ‘Anaheim’ pepper plants with *Pepper golden mosaic virus* (PepGMV) DNA-A mosaic strain, pMoA, and PepGMV distortion strain, pDA, and the DNA-B chimeras, respectively, 14–15 days post-inoculation.

DNA-A	DNA-B Chimeras	Symptoms
pDA	pDB:MoBC1 (MoBC1 gene in DB wild-type)	mosaic
pDA	pDB:MoBC1ORF (MoBC1 ORF in DB wild-type)	distortion
pDA	pDB:MoBC1 Prom (MoBC1 promoter in DB wild-type)	mosaic
pMoA	pMoB:DBC1(DBC1 gene in MoB wild-type)	distortion
pMoA	pMoB:DBC1ORF (DBC1 ORF in MoB wild-type)	mosaic
pMoA	pMoB:DBC1Prom (DBC1 promoter in MoB wild-type)	distortion

## Data Availability

All data collected, described, and reported for this manuscript are presented here in figure or table format.
